# Monthly Summary

**Published:** 1878-10

**Authors:** 


### MONTHLY SUMMARY.

*Nature and Treatment of Neuralgia.*—Dr. James B. Baird, of Atlanta, Georgia, in a paper on this subject, read before the Georgia Medical Association, sets forth the following conclusions:

1. Neuralgia, literally considered, is not a disease, but a symptom of some unknown pathological state of a sensory nervous trunk, fibre or filament.

2. It is characterized by attacks of pain of greater or less intensity, frequency and duration.

3. The pain is paroxysmal, being marked by more or less complete and protracted remissions or intermissions.

4. The causes of neuralgia are numerous, but the most frequent cause, in the opinion of the writer, in this country, at least, is malaria.

5. Its essential pathological nature is unknown

6. The diagnosis as to the existence of the affection is ordinarily sufficiently easy, though the attempt to trace its origin, is frequently attended with much difficulty.

7. The prognosis depends upon the cause and our ability to remove it by the means of treatment at our disposal.

8. The treatment should be constitutional and local.

9. The general treatment should be directed, as far as our knowledge or suspicions will warrant, against the cause of the attack. It should be persevered in with a view of removing any supposed taint or infection that may exist, and of augmenting the powers of the system.

10. Apart from any constitutional therapeutic indications, electricity and morphia hypodermically are the most reliable remedies that we possess.

11. Galvanism is by far the most efficient form in which electricity can be administered.

12. To secure the best attainable results, galvanization must be used intelligently, and, in many cases, perseveringly.—*Pacific Medical and Surgical Journal.*

---

*Chloral Hydrate Locally Applied in Tetanus.*—Dr. Bigelow reports, in the *Practitioner*, a case of tetanus caused by a rusty nail penetrating the foot, which was relieved in less than twenty minutes by introducing a drachm of chloral hydrate into the wound, after it had been enlarged by incision.—*Canada Lancet.*

*Treatment of Wounds.*—The most simple application for sealing up wounds is the old-fashioned tincture of benzoin, and it is the most successful. By it nearly all flesh wounds heal rapidly, while they do not do so under watery and fatty dressings. Tincture of benzoin has a remarkable property of uniting tissues and combining with blood. It is antiseptic, and assisted by cotton-wool pads of lint and firm bandaging, will arrest hemorrhage from all vessels less in size than the radial artery. Non-recent wounds which suppurate it is not desirable to heal by adhesion. The most important item in the treatment of these is ventilation with as pure air as possible. None but the most evil results follow the application of waterproof materials such as oiled silk and gutta percha tissue over the dressings. Such wounds invariably stink and slough; the wound is made unduly hot, products of decomposition are retained, the surface has a grayish, grumous aspect and loses substance daily. A simple piece of lint or muslin covered by cerate, or dipped in lotions of Condy's fluid (1 to 40), or tincture of myrrh and water (1 to 20), spirit and water or weak carbolic acid lotion (1 to 60), with just a layer of bandage to retain the dressing in its place, is all that is necessary, save a daily syringing and washing with warm Condy's fluid and water. (Mr. Phillip Cowen, p. 124.)—*Braithwaite's Retrospect of Practical Med. and Surg.*

---

*Hypodermic Injections of Chloroform.*—These injections have been highly recommended by eminent physicians as a substitute for morphine. It is claimed that they are painless, that they relieve pain rapidly and for several hours, and that they are entirely innocuous, being followed by neither local nor general symptoms. Dr. Jochheim, of Darmstadt, however, reports a case in which the injection of only ten drops of chloroform, which is only one half what is frequently administered, was followed by severe local disturbances. Five hours after the injection a violent local inflammation set in, and in twenty-four hours a hard, black slough had formed which was not cast off by suppuration until six weeks afterwards.—*Allg. Med. Cent. Zeit.*

---

*Sulphate of Quinine.*—A property of Sulphate of Quinine not well known.—This property consists in the modification it causes on suppurating surfaces when it is applied locally. The injection of a solution of 60 centigrammes of sulphate of quinine in 60 to 100 grammes of distilled water is very advantageous in the treatment of empyema. The same injection is efficacious in gonorrhœa, and an ointment of sulphate of quinine exercises a cicatrizing action on wounds and chronic ulcers. The injections of quinine have the same action on suppurating cavities and fistulous tracts.—*Gazetta Medica Italiania.*

*A Rival to Carbolic Acid.*—Prof. Volkmann, of Halle, who has achieved such brilliant results with the use of Lister's method in surgery, has adopted the new antiseptic, thymol, in his clinics. His assistant, Dr. Ranke, reports fifty-nine operations in which the thymol was used in place of carbolic acid, with strikingly good results. These operations included several amputations—of the leg, arm, breast, and foot; four excisions of the elbow; a gunshot wound of the knee-joint; a secondary amputation of the thigh; an excision of the hip, one of the shoulder, etc. The results obtained thus far in the major operations show that, under thymol, the secretion is much less and the rate of healing much quicker than when carbolic acid is used. Thymol has the advantage of being innocuous and non-irritant, and of not causing the least anæsthesia of the skin. The solution used consisted of thymol 1 gramme, alcohol 10, glycerine 20, and water 1,000 grammes. The much greater expense of thymol is counter-balanced, Dr Ranke maintains, by the smaller quantity required and the few bandages needed.—*New York Medical Journal.*

---

*Salicylic Acid and Borax.*—It may be interesting and perhaps useful for some readers of the *Journal* to know that while a solution containing ten grains of salicylic acid and ten grains of borax in one ounce of water has a very bitter taste and an acid reaction, a solution containing ten grains of salicylic acid and fifteen grains of borax has no disagreeable taste, and is nearly neutral. This solution appears to possess all the valuable properties of salicylic acid, and forms an agreeable means of using the acid internally or as a gargle.—*London Pharm. Jour.*

---

*Abscess of Lower Lip Attributed to Poisoning by Paris Green.*—Dr. R. Wilson, of Washington County, N. Y., writes us: The ravages of the potato bugs in this section have caused the farmers to use an immense quantity of Paris Green, to destroy them. About a week since a young man, by the name of Albert Calkins, picked an apple from the ground, where it lay among potato vines upon which "Paris green" had been used, and ate it, or rather sucked the juice. Shortly his lower lip began to inflame, and after acute suffering an abscess formed, which, on being opened, discharged a large amount of pus. Abscess of the lip is not, I believe, of common occurrence.

Paris Green is said to be a compound of arsenic and copper. Of Scheele's Green, Dr. Taylor says that it prdouced, in a watchmaker, "swelling and ulceration of the lips."—*Med. and Surg. Reporter.*

*Antiseptic Solution of Benzoic Acid.*—A handy preparation for dressing and disinfecting wounds, suggested by the experiments of Professor Salkowski, of Berlin, on the strongly antiseptic properties of benzoic acid, has been found very valuable and pleasant for its deodorizing effects in practice on shipboard by Dr. Senftleben. It is a solution of half an ounce of pure *acidum benzoicum e resina* in ten ounces of rectified spirit. This preparation may be used like the alcohol phenique of the French, but is of far more agreeable smell and taste. It may be mixed with water for making lotions, gargles, mixtures, and will probably be found very effectual in naval and military practice. It may also become an article of the toilet; for, like salicylic acid, benzoic acid is innocuous, but its antiseptic action is by some considered to be stronger, and it volatilizes practically almost as readily as carbolic acid, but with a very different odor.—*Pharm. Journal.*

---

*On the Combined Use of Chloroform and Morphia.*—Professor Kœnig, in a communication to the *Centralblatt fur Chirurgia,* (No. 39, 1877,) says he has combined the hypodermic administration of morphia with that of chloroform in a large number of cases, with very favorable results. It is seldom necessary to give more than one or at most two centigrammes [one-sixth to one-third grain.]

The indications for the use of morphia during chloroform-narcosis are twofold: 1. Motor disturbances occurring before or during chloroform-inhalation unless these are very transitory: 2. Operations of such a nature that the chloroform-narcosis cannot be maintained throughout, and especially towards the end. Among the latter may be particularly mentioned operations upon the eye, plastic operations, extirpation of tumours from the soft parts of the face. The object of using morphia is to induce analgesia over and above the chloroform-narcosis, and also that this narcosis should not be pushed so far. As regards any danger which may be connected with the combination of narcotics, Kœnig esteems this lightly. He says that out of seven thousand cases in which he has used chloroform, none have died from it, and many of these took morphia also.

---

*The Prevention of Chloroform Narcosis.*—In the *Vierteljahrscrift fur Gericht. Med.* Dr. Wachsmuth, of Berlin, makes the important statement that if one-fifth, part of oil of turpentine is added to chloroform, the latter can be administered to the fullest anæsthesia without the slightest risk, as the turpentine prevents, by its stimulating properties, the pulmonic paralysis, which is the proximate cause of death in fatal chloroform narcosis.—*Med. and Surg. Reporter.*

*Abortive Treatment of Furunculus.*—Dr. Lieven observed at the Petersburg Medical Society [*Petersburg Med. Woch.*, Dec. 29] that all modes of treatment hitherto tried [such as early incision, cauterizing, and cold or warm applications] have failed to arrest the further development of furunculus that has once commenced. The following procedure, however, brings it to a stand: A burning, pricking, itching, suddenly occurring in a normal portion of the skin, announces the commencement of the development of the furunculus, and on the same day a small and quite superficial induration can be felt at the spot. If the skin be now superficially scraped with a small knife, so that a drop or two of blood may be pressed through the epidermis, no furunculus will be developed. This result would seem to show that the affection originates in the uppermost layer of the corium, and perhaps in the capillaries of the papillæ and not, as hitherto received, in the subcutaneous connective tissue, with succeeding necrosis of the corium and epidermis. Disturbance of the digestive organs [frequently diarrhœa] always precedes or accompanies furunculus; but a plethoric or decrepid constitution is no necessary condition, as it may occur in one that is quite normal.—*Med. Times and Gazette.*

---

*A New, Cheap and Self-Generating Disinfectant.*—Under this title Dr. John Day, of Geelong, Australia, recommends for use in civil and military hospitals, and also for the purpose of destroying the poison-germs of small-pox, scarlet fever, and other infectious diseases, a disinfectant ingeniously composed of one part of rectified oil of turpentine and seven parts of benzine, with the addition of five drops of oil of verbena to each ounce. Its purifying and disinfecting properties are due to the powder which is possessed by each of its ingredients of absorbing atmospheric oxygen and converting it into peroxide of hydrogen—a highly active oxidizing agent. and very similar in its nature to ozone. Articles of clothing, furniture, wall-paper, carpeting, books, newspapers, letters, etc., may be perfectly saturated with it without receiving the slightest injury; and when it has been once freely applied to any rough or porous surface, its action will be persistent for an almost indefinite period. This may, at any time, be readily shown by pouring a few drops of a solution of iodine of potassium over the material which has been disinfected, when the peroxide of hydrogen, which is being continually generated within it, will quickly liberate the iodine from its combination with the potassium and give rise to dark brown stains. It may be applied with a brush or a sponge, or, if more convenient, as the case with certain articles, such as books, newspapers and letters, it may be simply poured over them until they are well soaked; they may then be allowed to dry either in a warm room or in the open air.—*Brit. Med. Jour.*

*Howard's Method of Artificial Respiration.*—Dr. Benjamin Howard, U. S. A., has been attracting considerable attention in London by his demonstration of his method of artificial respiration. As some years have passed since it was first laid before the profession, we give anew the process he recommends :—

In order to dispose of the accumulations in the stomach or chest, the patient, stripped to the waist, is turned face downward, and a firm bolster being placed beneath the epigastrium, makes that the highest and the mouth the lowest point. Pressure being made on the back the object is accomplished by both ejection and drainage. The patient is quickly turned upon his back, the bolster placed beneath it, making again the epigastrium and the anterior margins of the costal cartilages the highest points of the body, the hips, shoulders, and occiput barely resting on the ground. The patient's wrists are seized, and, the utmost possible extension being secured with them crossed behind his head, they are pinned to the ground with the left hand, so as to maintain it. With the right thumb and forefinger, armed with the corner of a dry handkerchief, the tip of the tongue is withdrawn and held out of the extreme right corner of the mouth. [The wrists and tongue may be confined to any aid.] In this position two-thirds of the entrance to the mouth is free. The epiglottis, by this backward curvature of the neck, is precluded from pressure by undue flexion. The head is dependent, the free margins of the costal cartilages are as prominent as they can be made. The epigastrium being the highest point, the diaphragm is neither embarrassed from pressure above or from below. To produce respiration the operator kneels astride the patient's hips and rests the ball of each thumb upon the corresponding costo-xiphoid ligaments, the fingers falling naturally into the lower intercostal spaces. Resting his elbows against his sides, and using his knees as a pivot, the operator throws the whole weight of his body slowly and steadily forward, until his mouth nearly touches the mouth of the patient, and while one might slowly count one, two, three; then, suddenly, by a final push, he springs back to his first position on his knees, remaining there while one might slowly count one, two; then repeat, and so on about eight or ten times a minute. The resiliency of the ribs insures an instant rebound to the point of departure. The operation can be practiced by anybody, anywhere, before or after division of the funis; in a bath, bed or boat; and friction, electricity, insufflation, or tracheotomy could be practiced simultaneously, without inconvenience.—*Med. and Surg. Reporter.*

